# Prognostic significance of infarct core pathology in ST-elevation myocardial infarction survivors revealed by non-contrast T1 mapping cardiac magnetic resonance

**DOI:** 10.1186/1532-429X-17-S1-O26

**Published:** 2015-02-03

**Authors:** David Carrick, Caroline Haig, Samuli M Rauhalammi, Nadeem Ahmed, Ify Mordi, Margaret McEntegart, Mark Petrie, Hany Eteiba, Stuart Hood, Stuart Watkins, Mitchell Lindsay, Ahmed Marous, Aleksandra Radjenovic, Ian Ford, Niko Tzemos, Keith G Oldroyd, Colin Berry

**Affiliations:** 1 Golden Jubilee National Hospital, Clydebank, UK; 2 Robertson Center for Biostatistics, University of Glasgow, Glasgow, UK; 3 Institute of Cardiovascular & Medical Sciences, University of Glasgow, Glasgow, UK

## Background

Myocardial longitudinal relaxation time (T1, ms) is a fundamental magnetic property of tissue that is related to water content and mobility. The pathophysiological and prognostic importance of native myocardial T1 values in acute ST-elevation myocardial infarction (STEMI) patients is unknown. We aimed to assess the clinical significance of infarct core native T1.

## Methods

We performed a prospective single center cohort study in reperfused STEMI patients who underwent CMR 2 days and 6 months post-MI. Native T1 CMR (MOLLI investigational prototype sequence: 3 (3) 3 (3) 5) was measured in myocardial regions-of-interest. The infarct territory and microvascular obstruction (MVO) were depicted with late gadolinium enhancement CMR. Adverse remodeling was defined as an increase in LV end-diastolic volume (LVEDV) ≥ 20% at 6 months. All-cause death or heart failure hospitalization was a pre-specified outcome that was assessed during follow-up.

## Results

300 STEMI patients (mean±SD age 59±12 years, 74% male, 114 with anterior STEMI) gave informed consent and had CMR (14 July 2011 - 22 November 2012). Of these, 288 STEMI patients had evaluable T1 maps. Infarct size was 18 ±14% of LV mass. One hundred and forty five (50%) of 288 patients had late MVO, whereas 160 (56%) patients had infarct core pathology revealed by native T1. Native T1 within the infarct core (996.9±57.3; p<0.01) was higher than in the remote zone (961±25 ms; p<0.01) but lower than in the area-at-risk (1097 ±52 ms). In multivariable linear regression, native T1 in the infarct core was negatively associated with age, initial systolic blood pressure, TIMI coronary flow grade at initial angiography, Killip class at presentation and neutrophil count (all p<0.05), independent of LVEF, LVEDV or infarct size.

At 6 months, LVEDV increased by 5 (25) ml (n=262 patients with evaluable data). Adverse remodeling occurred in 30 (12%) patients and 23 (76.7%) of these patients MVO at baseline. T1 in the infarct core was a multivariable predictor of adverse remodeling (-0.01 (-0.02, -0.00); p=0.048).

288 (100%) patients were followed-up for a median of 845 days. Thirty (10.4%) patients died or experienced a heart failure event and 13 (4.5%) of these patients experienced the event post-discharge. Infarct core native T1 predicted all-cause death or heart failure post-discharge (hazard ratio 0.969, 95% CI 0.953, 0.985; p<0.001) including after adjustment for LVEF (p<0.001) and LVEDV at baseline (p<0.001), and was comparable with MVO.

## Conclusions

Infarct core native T1 represents a novel alternative non-contrast imaging biomarker for infarct characterization and prognostication in STEMI survivors.

## Funding

N/A.

**Figure 1:**
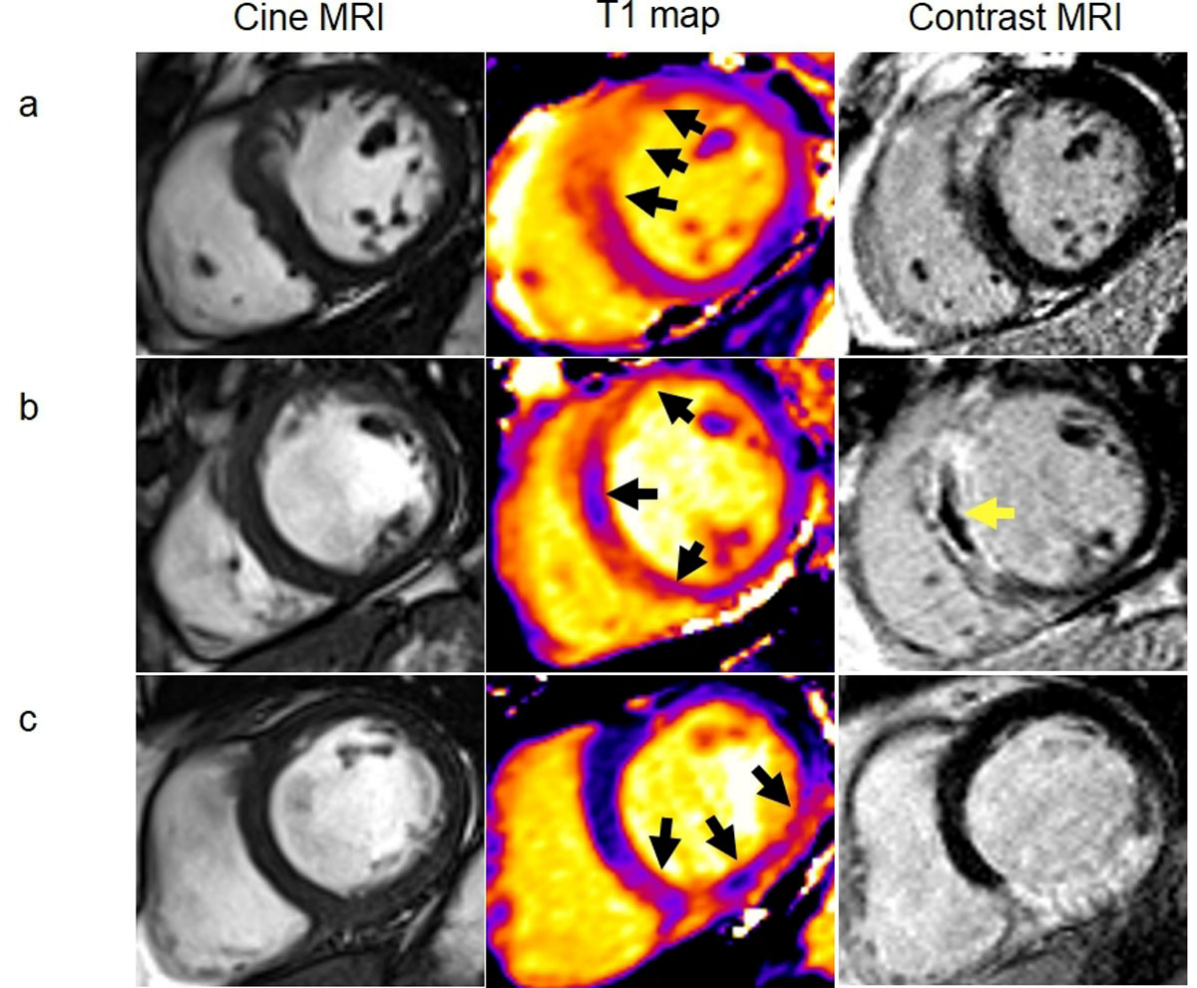
Three patients with acute STEMI treated by primary PCI. Cardiac MRI was performed for each patient 2 days later. (a) *Patient with no T1 hypointense infarct core and no microvascular obstruction.* Six month follow-up MRI revealed final infarct size was 15.6% of left ventricular mass and left ventricular end-diastolic volume (LVEDV) reduced was reduced vs. baseline scan. This patient had an uncomplicated clinical course. (b) *Patient with both T1 hypointense infarct core and microvascular obstruction.* Six month follow-up MRI revealed final infarct size was 22.6% of left ventricular mass and an increased LVEDV. During follow-up this patient was hospitalized for new onset heart failure. (c) *Patient with T1 hypointense infarct core*, *but no microvascular obstruction.* Six month follow-up MRI revealed final infarct size was 22.1% of left ventricular mass and a significantly increased LVEDV. No adverse events during follow-up.

